# Improved indocyanine green retention after short-term lenvatinib withdrawal in three patients with hepatocellular carcinoma

**DOI:** 10.1007/s12328-021-01470-y

**Published:** 2021-06-27

**Authors:** Rie Sugimoto, Hiroki Inada, Yuki Tanaka, Takeshi Senju, Yoshifusa Aratake, Akira Nakanishi, Masami Miki, Lingaku Lee, Terumasa Hisano, Yoshihiro Matsumoto, Yohei Mano, Tomohiro Iguchi, Keishi Sugimachi, Yukihiko Okumura, Kenichi Taguchi, Masayuki Furukawa

**Affiliations:** 1 Department of Hepato-Biliary-Pancreatology, National Hospital Organization Kyushu Cancer Center, Fukuoka City Fukuoka Prefecture, 811-1395 Japan; 2 Department of Hepato-Biliary and Pancreatic Surgery, National Hospital Organization Kyushu Cancer Center, Fukuoka City Fukuoka Prefecture, 811-1395 Japan; 3 Department of Pathology, National Hospital Organization Kyushu Cancer Center, Fukuoka City Fukuoka Prefecture, 811-1395 Japan

**Keywords:** Hepatocellular carcinoma, Conversion surgery, Lenvatinib, Indocyanine green retention test

## Abstract

Use of lenvatinib, which has a high response rate in advanced hepatocellular carcinoma, sometimes results in tumor shrinkage and resectability of previously unresectable liver cancers. In Asia, including Japan, liver reserve, one of the determinants of resectability, is mainly determined by the indocyanine green (ICG) retention rate. Three patients with advanced liver cancer treated at our institution had very poor ICG retention rates during treatment with lenvatinib. Lenvatinib may reduce blood flow in both cancerous and non-cancerous regions by inhibiting vascular endothelial growth factor. Therefore, accurate determination of liver function likely requires withdrawal of this treatment several days before ICG retention testing.

## Introduction

Lenvatinib was approved as a first-line treatment after a head-to-head trial with sorafenib, which had previously been the standard of care in accordance with the findings of the REFLECT study [1]. Lenvatinib, a multikinase inhibitor, inhibits vascular endothelial growth factor receptor (VEGFR) 1–3, rearranged during transfection proto-oncogene (RET), fibroblast growth factor receptors (FGFR) 1–4, and platelet-derived growth factor receptor (PDGFR) alpha [2] [3] It has a high response rate [1]. Since the introduction of lenvatinib, previously unresectable hepatocellular carcinoma has become suitable for conversion surgery in some cases [4]. A preoperative indocyanine green (ICG) retention test, in which ICG is administered intravenously, enables assessment of hepatic reserve because the rate of retention of ICG reflects effective hepatic blood flow and the ability of hepatocytes to capture and excrete foreign substances into bile. The ICG retention test is widely used to determine whether liver resection is indicated, especially in Asia [5]. However, the effect of lenvatinib on ICG retention rate has not been reported. In this study, we report three patients with such poor ICG retention rates that surgery was considered contraindicated despite tumor shrinkage by lenvatinib. In these cases, when the ICG retention rate was reassessed several days after lenvatinib withdrawal, it had improved to the degree that these patients’ tumors were deemed suitable for conversion therapy. These three cases demonstrate changes in ICG retention rate after cessation of lenvatinib.

## Case report

### Case 1

**Fig. 1:**
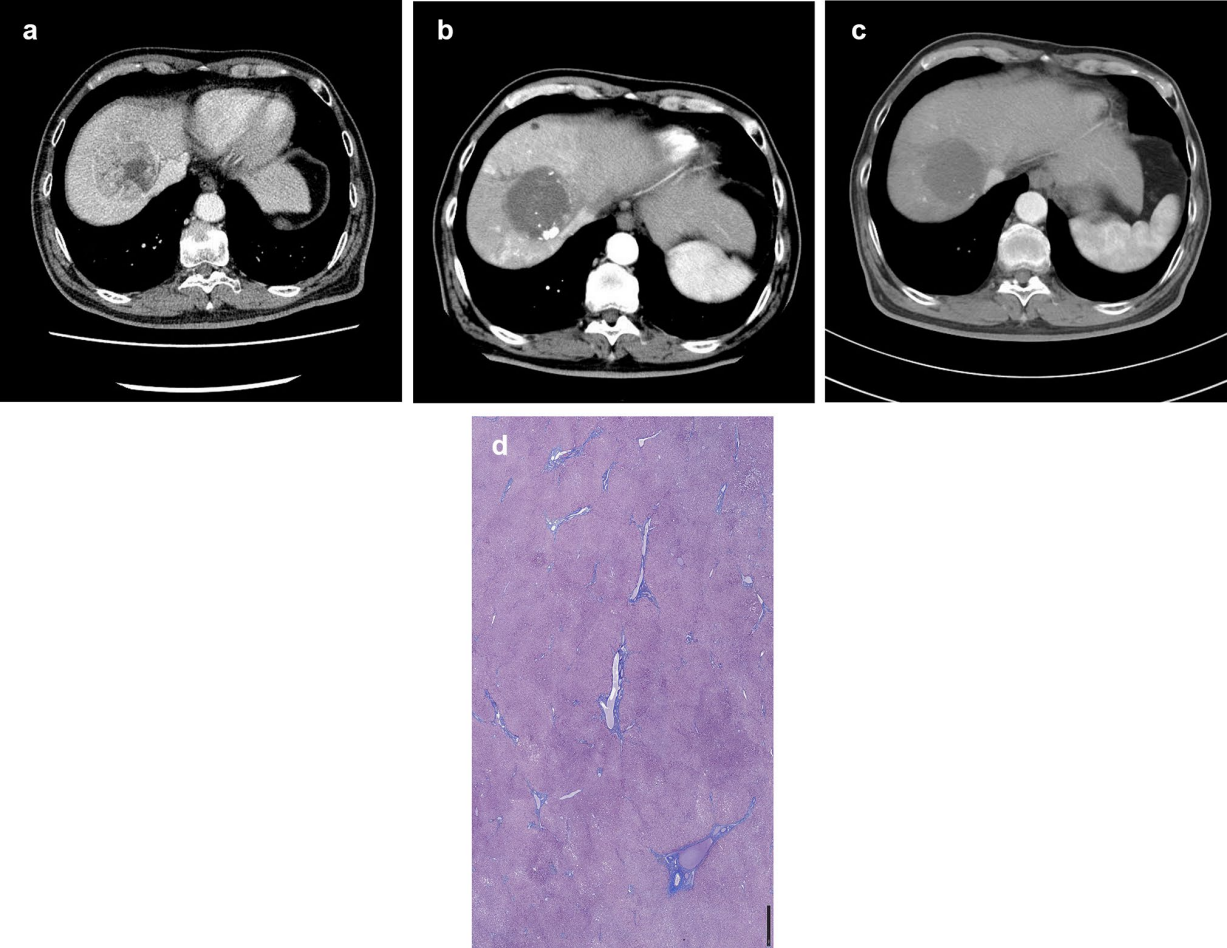
Case 1 **a** Contrast-enhanced CT on presentation. **b** Contrast-enhanced CT before commencement of lenvatinib treatment. **c** Contrast-enhanced CT at the time of ICG retention testing. **d** Photomicrograph of the resected specimen. Masson trichrome staining (non-cancerous area)

A 62-year-old man was admitted to our hospital for liver cancer treatment. He had been diagnosed with hepatitis C at age 50 and received direct-acting antiviral (DAA) treatment 3 years prior to being referred to us, achieving a sustained virological response (SVR). He continued to attend an outpatient clinic for treatment of his diabetes and was diagnosed with liver damage just prior to referral to our department for management of a 6-cm hepatocellular carcinoma in S4/8 of the liver. A computed tomography (CT) scan revealed a 6.8-cm high–low pattern mass in liver S4/8 (Fig. 1a) located at the confluence of the inferior vena cava (IVC) and right and middle hepatic veins. The portal and hepatic veins were compressed, but not infiltrated, by the tumor, which was considered too large to be excised. The patient underwent two drug-eluting beads-transcatheter arterial chemoembolization (DEB-TACE) and conventional TACE (cTACE). However, residual liver cancer prompted initiation of lenvatinib 12 mg 1 month after performing cTACE (Fig. 1b). His Child–Pugh category was A and modified albumin–bilirubin (mALBI) grade 2a (score − 2.562) at the start of lenvatinib treatment. His relative dose intensity (RDI) during the treatment was 100%. Twelve months after commencement of lenvatinib, the liver cancer no longer showed a clear contrast effect (Fig. 1c) and no portosystemic shunts were detected by imaging. We considered performing a liver resection for conversion purposes. When measured during lenvatinib treatment, the ICG retention rate was 34.8% despite a Child–Pugh score of 5 and a mALBI Grade of 2a (score − 2.502) (Table 1). The mALBI grade did not worsen during treatment; however, the ICG retention rate was so poor that planned surgery was deferred. When the ICG retention rate was reassessed 5 days after lenvatinib withdrawal, it was found to have improved by 22.6%. At that time, the 99mTc-galactosyl serum albumin (^99m^Tc-GSA) liver scintillation was HH15 = 0.766 and LHL = 0.912, and ^99m^Tc-GSA equivalent predicted an ICG retention rate of 12.8%. This rate had improved to 14.8% by Day 9 after lenvatinib withdrawal. The patient was then considered to have adequate liver reserve and underwent a central two-area resection on Day 11 after drug withdrawal. He was discharged from hospital after a good postoperative course and remained alive with no recurrence 9 months later. The resected tumor was a moderately to poorly differentiated hepatocellular carcinoma with extensive necrosis and micro-portal vein tumor thrombosis. The fibrosis in the non-cancerous area was relatively minor, and the liver was pre-cirrhotic, not cirrhotic. (Fig. 1d).

**Table 1:** Clinical data at ICG retention rate

	Case1	Case2	Case 3
Child–Pugh (score)	A (5)	A (6)	A (6)
Alb (g/dl)	3.9	3.3	3.2
T.Bil (mg/dl)	1.0	1.1	1.0
PT (%)	82	114	93
mALBI	G2a	G2b	G2b
ICG retention rate (under treatment) (%)	34.8	38.6	32.2
ICG retention rate (withdrawal) (%)	14.8	19.2	24.2
Withdrawal period (day)	9	5	3
ICG reduction rate per day of Len withdrawal (%)	2.2	3.88	2.6
Lenvatinib treatment period (month)	13	13	6
AFP (ng/ml)	4	7	10,381
PIVKA-II (mAU/ml)	78	33	505,740

### Case 2

**Fig. 2:**
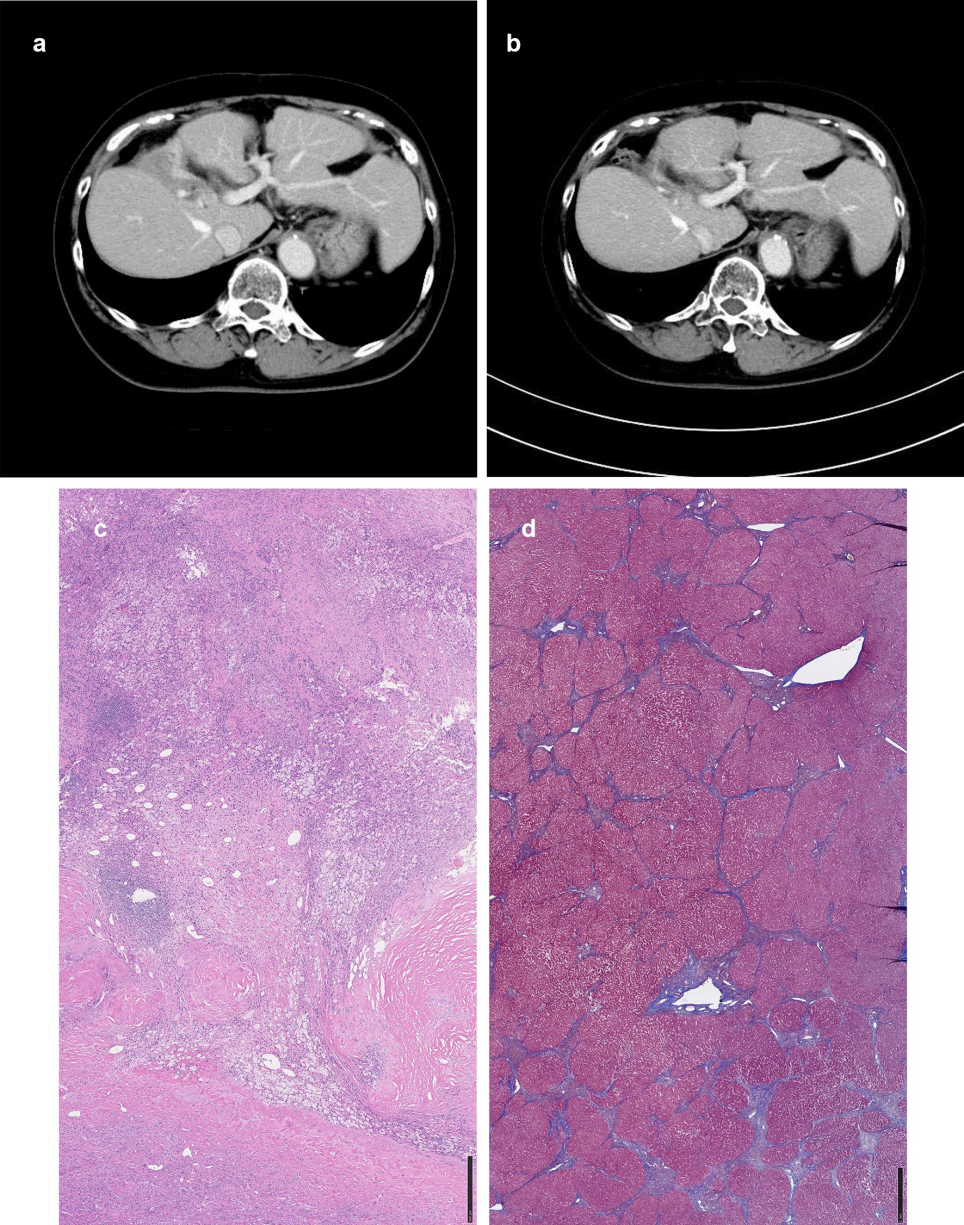
Case 2 **a** Contrast-enhanced CT before commencement of lenvatinib treatment. **b** Contrast-enhanced CT at the time of ICG retention testing. **c** Photomicrograph of the resected specimen (cancerous area) **d** Photomicrograph of the resected specimen (non-cancerous area). Masson trichrome staining

A 77-year-old woman who had previously been noted to have hepatitis C but had not been treated with interferon or other treatments was admitted to our hospital for management of multiple liver cancers. On physical examination, she was 148 cm tall, weighed 54 kg, and had a performance status (PS) score of 0. She underwent TACE and radiofrequency ablation (RFA) during the year of referral and partial resection the subsequent year. Pathological examination revealed a moderately to poorly differentiated hepatocellular carcinoma. She was treated for hepatitis C the following year, achieving a SVR with oral DAA. However, 8 weeks after commencing that treatment, a hepatocellular carcinoma extending into the bile ducts was detected. After insertion of a bile duct stent, she underwent radiation therapy (intensity-modulated radiation therapy; 50 Gy). Recurrence was detected 6 months later (Fig. 2a), at which time the ICG retention rate was 17.2% and the mALBI grade G2a (score − 2.480). Oral lenvatinib was started at 8 mg. Development of proteinuria, edema, hand–foot syndrome, and fungal infection necessitated dose reduction and she continued lenvatinib at 4 mg for six doses with 1 day off. Her average RDI was 48%. Thirteen months after commencement of lenvatinib, the bile duct stenosis had improved and we considered liver resection for conversion (Fig. 2b). At that time, no portosystemic shunts were detected on imaging. Her Child–Pugh score was 5 points and her mALBI grade G1 (score − 2.786). There was no increase in mALBI score during treatment despite the patient's ICG retention rate being poor (38.6%). Therefore, surgery was postponed and lenvatinib continued (Table 1). One month later (14 months after starting lenvatinib), the ICG retention rate was measured 5 days after withdrawal of lenvatinib, at which time there was an improvement of 19.2% (Table 1). ^99m^Tc-GSA liver scintillation 6 days after withdrawal was HH15 = 0.741 and LHL = 0.906, with ^99m^Tc-GSA equivalent predicting an ICG retention rate of13.4%. The patient's liver function was then deemed adequate for liver resection. However, lenvatinib was continued because there was a waiting list for surgery. One month later (15 months after commencing lenvatinib and 5 days after its withdrawal), the ICG retention rate was measured again and found to be 33%. Surgery was, therefore, rescheduled and lenvatinib suspension continued. One month later, the ICG retention rate had dropped to 27% and liver right lobectomy and biliary reconstruction were performed. There was no residual tumor in the resected specimen, a nodular lesion showing only massive aggregates of foamy histiocytes with mild to moderate chronic inflammatory infiltration (Fig. 2c). Non-cancerous areas showed focal bridging fibrosis (between P and P) and mild fibrous enlargement of the portal vein area (Fig. 2d). The patient’s postoperative course was good, no liver failure occurred, and no recurrence was detected 7 months after surgery.

### Case 3

**Fig. 3:**
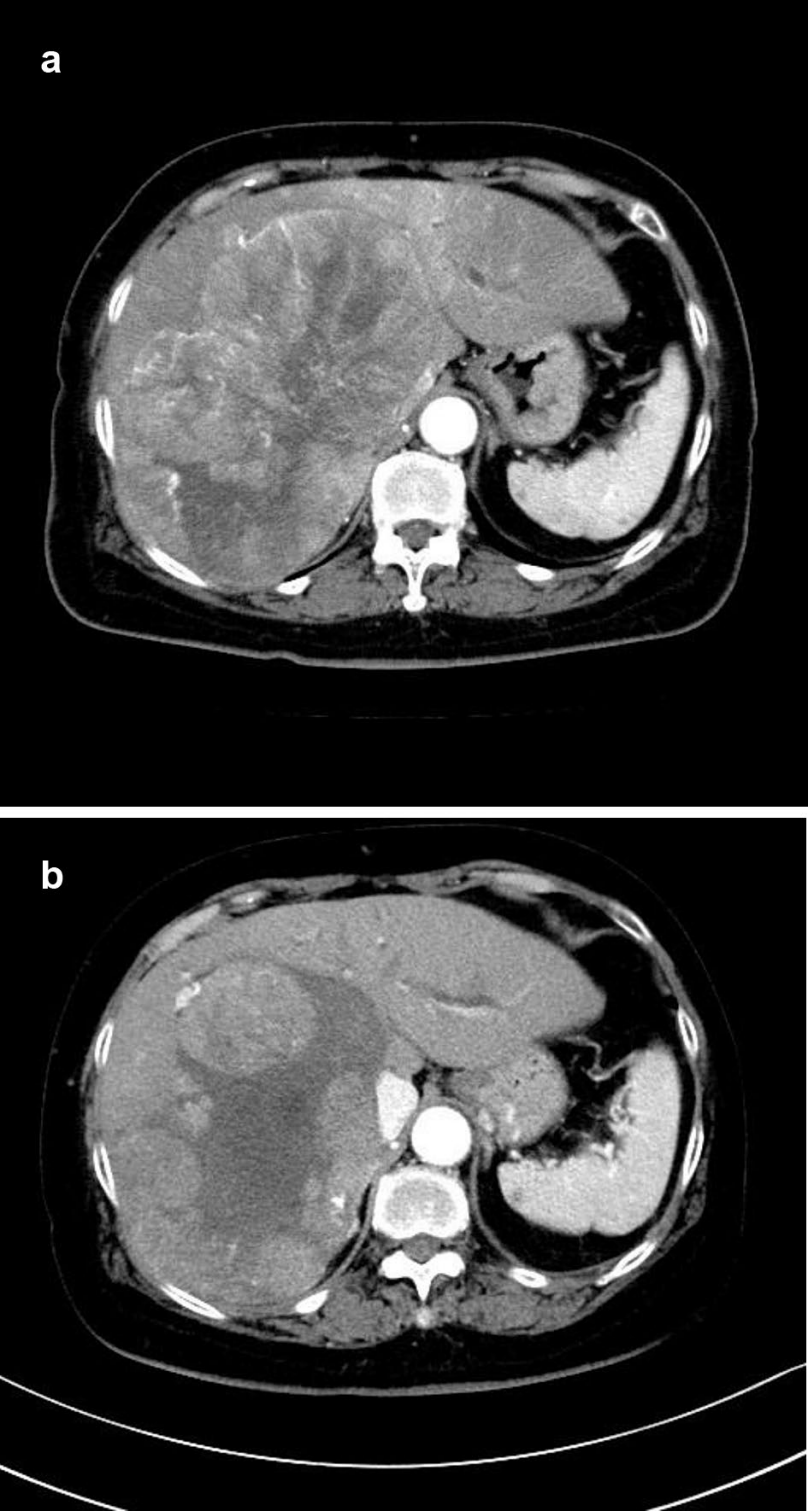
Case 3 **a** Contrast-enhanced CT before commencement of lenvatinib treatment. **b** Contrast-enhanced CT at the time of ICG retention testing

Five months after a 76-year-old woman attending a clinic for diabetes was diagnosed with liver damage, she was diagnosed as having a giant liver cancer and referred to our hospital. Imaging (Fig. 3a) revealed a 15-cm large high–low pattern mass in the right lobe of the liver. The portal and hepatic veins were blocked, but not infiltrated, by the tumor. Lenvatinib 8 mg was started. Child–Pugh category was A and mALBI grade 1. Her RDI during treatment was 100%. The hepatocellular carcinoma initially shrank slightly but was found to have enlarged 6 months after commencing lenvatinib) (Fig. 3b). In addition, urine protein was increased as an adverse effect of lenvatinib, necessitating review of her treatment (Fig. 3b). At that time, serum albumin was a little low because of the amount of urinary protein and her mALBI score was worse than at the beginning of treatment (G1 → G2b). At that time, no portosystemic shunts were detected on imaging. Despite a Child–Pugh score of 5 points and mALBI grade 2b, the ICG retention rate was 32.2% during continuation of oral lenvatinib. However, three days after ceasing lenvatinib, the ICG retention rate had improved to 24.6% (Table 1). After addition of DEB-TACE, she was switched to tyrosine kinase inhibitors (TKIs) because of detection of lung metastasis.

## Discussion

The ICG retention test is widely used in Asia to assess liver function and blood flow [6], this being a major criterion for liver resection (Makuuchi criteria) [7] [8]. Because ICG is not degraded elsewhere, being metabolized only in the liver and excreted in the bile, ICG retention accurately reflects liver metabolism. ICG, a synthetic dye, binds to blood lipoproteins and is transported to the liver, where it is ingested by hepatocytes during transit through the sinuses and excreted into the bile without undergoing conjugation. The amount of ICG transferred from blood to bile is determined mainly by effective hepatic blood flow and hepatocyte pigment uptake. Thus, the ICG retention rate reflects both effective hepatic blood flow and hepatocyte uptake capacity. It is thought that the use of ICG retention rate to determine the extent of liver resectability may reduce postoperative mortality [9]. It is, therefore, a major factor in determining eligibility for liver resection [10] [11].

Lenvatinib, a kinase inhibitor, exerts its antitumor effects by inhibiting angiogenesis, which is important for tumor growth and metastasis, mainly by inhibiting VEGFR1–3 and FGFR1–4. Lenvatinib also inhibits PDGFR alpha, stem cell factor receptor (KIT), and RET, receptor tyrosine kinases that have been reported to be involved in cancer progression [2] [3] [12]. Inhibition of these kinases is thought to have marked antitumor effects. However, multi-molecular target agents do not only affect vessels in neoplasms; they have also been found to damage the vascular structures of some healthy organs, such as the thyroid and adrenal glands and gastrointestinal tract [13] [14]. Iwamoto et al. found that lenvatinib administration inhibits blood flow in normal organs and that the vasculature of both tumors and organs fluctuates in parallel with administration and withdrawal of lenvatinib [15]. We here present three patients whose ICG retention rates improved after several rounds of lenvatinib withdrawal (Table 1), indicating that conversion surgery was feasible despite our initial erroneous conclusion, which was based on the poor ICG retention rate on lenvatinib, that it was contraindicated. There is no clear protocol for how long this agent should be withdrawn prior to conversion surgery. Some authors have reported withdrawal approximately 10 days before surgery [4], however, to the best of our knowledge, there are no reports of timing of ICG retention testing in relation to such withdrawal. Initially, we did not realize that withdrawal prior to ICG retention testing was necessary and performed these tests, while the patient was still receiving oral lenvatinib. In all three cases, there was no or mild worsening of mALBI during TKI treatment. Despite the relatively well-preserved Child–Pugh scores and mALBI grades, we were close to considering the tumors were inoperable because of the poor ICG retention rates. However, when we became aware of the possibility of disordered blood flow in non-cancerous tissues, we withdrew lenvatinib until it would have largely been cleared from the blood, by which time the ICG retention rate had improved. According to Iwamoto et al., blood flow to the thyroid and adrenal glands of mice improved 2 days after withdrawal of lenvatinib [15]. It has also been reported that, in patients with liver cancer treated with sorafenib, portal blood flow was reduced 2 weeks after treatment in the response group [16]. In our cases, ICG retention rate improved after a short period of drug withdrawal, this possibly being attributable to reduction of blood flow in non-cancerous areas in response to lenvatinib treatment and improvement in vascular structures soon after withdrawal. Performing contrast-enhanced ultrasonography before and after treatment would have determined whether portal flow actually decreased; however, we did not do this. ICG clearance depends on cellular uptake, hepatic blood flow, and biliary excretion [17]. In patients with cirrhosis, we tend to think that irreversible conditions, such as increased portal pressure at the sinusoidal level, decreased blood flow, and decreased number of hepatocytes as a result of progression of liver fibrosis, are responsible for deterioration in portal blood flow. In fact, it has been reported that ICGR15 correlates with the degree of morphological damage of the liver in patients with cirrhosis [18].However, it is important to note that the ICG elimination rate may worsen in the absence of irreversible changes when patients are given agents, such as molecular targeted drugs, that directly affect factors involved in regulation of vascular function. The degree of fibrosis in the resected non-cancerous tissue was relatively mild in Cases 1 and 2, which is consistent with the above findings. The required duration of withdrawal prior to testing has not yet been established. However, the half-life of lenvatinib in solid tumors and lymphomas is reportedly 28 to 35 h [19]. Additionally, higher plasma concentrations of lenvatinib have been observed in Japanese patients with liver cancer than in those with other solid tumors at 24 h [20]. We therefore consider that a withdrawal period of at least 3 days is desirable. The improvements in ICG retention rates varied between our patients. It occurred relatively rapidly in Case 3, who had been on lenvatinib for a shorter time. Although Cases 1 and 2 had both been taking it for 13 months, improvement was smaller and slower in Case 1, in whom the RDI was maintained, and much faster in Case 2, in whom the RDI was not maintained. This may indicate that lenvatinib-induced impairment of blood flow is greater and less readily reversed in patients with preserved RDI. In Case 2, the improvement in ICG retention rate was smaller and slower after further treatment with lenvatinib, at which time the tumor was completely necrotic. Non-cancerous areas may take longer to recover when the tumor blood flow has been inhibited to the point of complete necrosis. Regardless, these findings suggest that lenvatinib should be withdrawn prior to performing an ICG retention test to determine whether a patient is eligible for conversion therapy. Without such withdrawal, liver function may be underestimated and the opportunity for conversion therapy missed.

In conclusion, when assessing ICG retention to determine whether conversion therapy is appropriate, prior withdrawal of lenvatinib results in a relatively rapid improvement in ICG retention rate. These findings suggest that lenvatinib impairs blood flow in non-cancerous areas and that blood flow improves after withdrawal of this drug. We recommend withdrawal of lenvatinib for several days before determining the preoperative ICG retention rate.

## References

[1] Kudo M, Finn RS, Qin S (2018). Lenvatinib versus sorafenib in first-line treatment of patients with unresectable hepatocellular carcinoma: a randomised phase 3 non-inferiority trial. Lancet.

[2] Matsui J, Yamamoto Y, Funahashi Y (2008). E7080, a novel inhibitor that targets multiple kinases, has potent antitumor activities against stem cell factor producing human small cell lung cancer H146, based on angiogenesis inhibition. Int J Cancer.

[3] Tohyama O, Matsui J, Kodama K (2014). Antitumor activity of lenvatinib (e7080): an angiogenesis inhibitor that targets multiple receptor tyrosine kinases in preclinical human thyroid cancer models. J Thyroid Res..

[4] Tomonari T, Sato Y, Tanaka H (2020). Conversion therapy for unresectable hepatocellular carcinoma after lenvatinib: three case reports. Medicine (Baltimore)..

[5] Makuuchi M, Sano K (2004). The surgical approach to HCC: our progress and results in Japan. Liver Transpl.

[6] Seyama Y, Kokudo N (2009). Assessment of liver function for safe hepatic resection. Hepatol Res.

[7] Makuuchi M, Kosuge T, Takayama T (1993). Surgery for small liver cancers. Semin Surg Oncol.

[8] Imamura H, Sano K, Sugawara Y (2005). Assessment of hepatic reserve for indication of hepatic resection: decision tree incorporating indocyanine green test. J Hepatobiliary Pancreat Surg.

[9] Wang YY, Zhao XH, Ma L (2018). Comparison of the ability of Child-Pugh score, MELD score, and ICG-R15 to assess preoperative hepatic functional reserve in patients with hepatocellular carcinoma. J Surg Oncol.

[10] Imamura H, Seyama Y, Kokudo N (2003). One thousand fifty-six hepatectomies without mortality in 8 years. Arch Surg..

[11] Kokudo T, Hasegawa K, Amikura K (2016). Assessment of preoperative liver function in patients with hepatocellular carcinoma—the albumin-indocyanine green evaluation (ALICE) grade. PLoS One..

[12] Nakamichi S, Nokihara H, Yamamoto N (2015). A phase 1 study of lenvatinib, multiple receptor tyrosine kinase inhibitor, in Japanese patients with advanced solid tumors. Cancer Chemother Pharmacol.

[13] Nakamura M, Zhang Y, Yang Y (2017). Off-tumor targets compromise antiangiogenic drug sensitivity by inducing kidney erythropoietin production. Proc Natl Acad Sci USA.

[14] Yang Y, Zhang Y, Cao Z (2013). Anti-VEGF- and anti-VEGF receptor-induced vascular alteration in mouse healthy tissues. Proc Natl Acad Sci USA.

[15] Iwamoto H, Suzuki H, Shimose S (2020). Weekends-off lenvatinib for unresectable hepatocellular carcinoma improves therapeutic response and tolerability toward adverse events. Cancers (Basel)..

[16] Hidaka H, Nakazawa T, Fujii S (2015). Early evaluation of response to sorafenib for hepatocellular carcinoma by duplex Doppler ultrasonography. Hepatol Res.

[17] Hunton DB, Bollman JL, Hoffman HN (1960). Studies of hepatic function with indocyanine green. Gastroenterology.

[18] Gu J, Zhang E, Liang B (2020). Effectiveness comparison of indocyanine green retention test with the cirrhotic severity scoring in evaluating the pathological severity of liver cirrhosis in patients with hepatocellular carcinoma and Child-Pugh grade A liver function. World J Surg Oncol.

[19] Dubbelman AC, Rosing H, Nijenhuis C (2015). Pharmacokinetics and excretion of (14)C-lenvatinib in patients with advanced solid tumors or lymphomas. Invest New Drugs.

[20] Ikeda M, Kobayashi M, Tahara M (2018). Optimal management of patients with hepatocellular carcinoma treated with lenvatinib. Expert Opin Drug Saf.

